# Recurrent Post-traumatic Morel-Lavallée Lesion of the Hip and Thigh Treated With Open Capsulectomy and Povidone-Iodine Sclerotherapy: A Case Report

**DOI:** 10.7759/cureus.86385

**Published:** 2025-06-19

**Authors:** Jung su Lee, Oh young Kwon, Jong soon Kim, Young hun Jung

**Affiliations:** 1 Orthopaedics, Hyosung City Hospital, Busan, KOR

**Keywords:** capsulectomy, chronic soft tissue injury, morel-lavallée lesion, posttraumatic seroma, povidone-iodine sclerotherapy, ultrasound-guided aspiration

## Abstract

Morel-Lavallée lesions (MLLs) are closed internal degloving injuries that may lead to persistent post-traumatic fluid collections. We report the case of a 65-year-old male patient with a recurrent MLL over the left hip and thigh following a fall. Initial management with aspiration and corticosteroid injection was unsuccessful, and open capsulectomy also failed to prevent recurrence. Due to the unavailability of conventional sclerosing agents, ultrasound-guided sclerotherapy using 10% povidone-iodine was performed. After two sessions, there was a marked reduction in the size of the lesion without complications. This case suggests that povidone-iodine may serve as a viable alternative sclerosant for recurrent MLLs when standard agents are inaccessible.

## Introduction

The Morel-Lavallée lesion (MLL) is a closed degloving injury caused by shearing forces that separate the hypodermis from the underlying fascia, creating a potential space filled with blood, lymphatic fluid, and necrotic fat [1,2]. Acute MLLs may resolve spontaneously, but chronic lesions often develop a fibrous pseudocapsule that hinders fluid resorption and resists conservative treatment [1,3].

For chronic MLLs, open capsulectomy with dead space closure is the standard approach, though recurrence may occur due to incomplete excision or extensive cavity formation [3,4]. When surgery fails or is unsuitable, sclerotherapy with agents like doxycycline offers a less invasive option [5-7]. However, injectable doxycycline is not always readily available.

Povidone-iodine, though less commonly used, can induce fibrosis via an inflammatory response and has been effective in treating postoperative seromas, particularly in breast surgery and pleurodesis [8-10]. Its role in MLL, however, remains underreported.

We present a case of chronic MLL in the hip and thigh treated initially with open capsulectomy, followed by staged povidone-iodine sclerotherapy due to recurrence. This case suggests povidone-iodine may serve as a practical alternative sclerosant for refractory MLLs.

## Case presentation

A 65-year-old male patient presented with left hip and thigh pain following a fall. Physical examination revealed extensive swelling and ecchymosis over the left gluteal and upper thigh region. Plain radiographs showed no fracture, but MRI revealed partial tears of the gluteus medius and minimus muscles with a sizable subcutaneous hematoma.

Initial management included analgesics and observation. By the third week post-injury, the patient returned with complaints of swelling and a fluid sensation. Fluctuation was noted on examination, and 110 cc of serous fluid was aspirated under ultrasound guidance, followed by triamcinolone injection. Symptoms temporarily improved but recurred after one week. Open surgical intervention was planned at four weeks post-injury due to recurrence and persistent collection.

**Figure 1:**
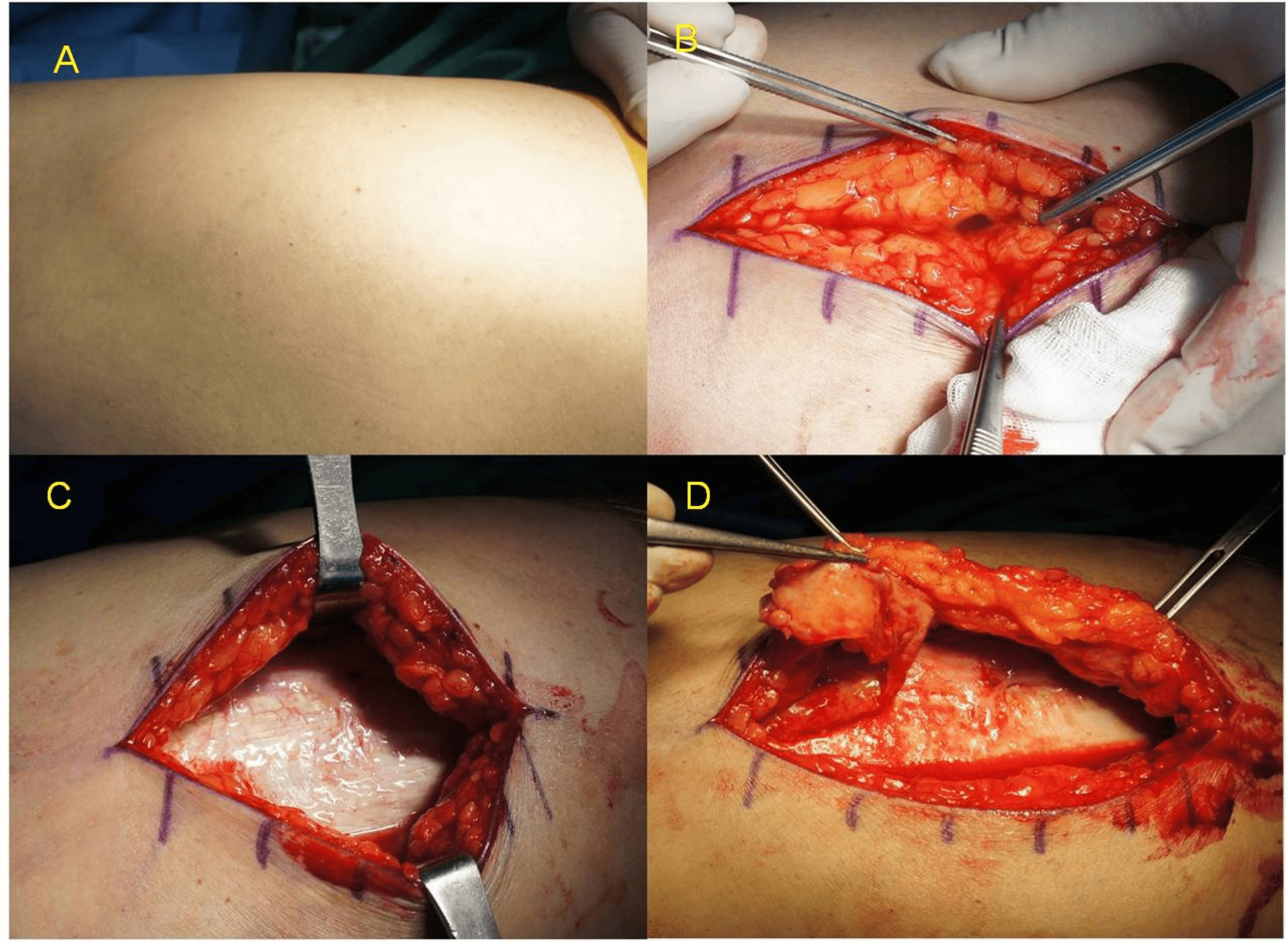
Intraoperative views of a chronic Morel-Lavallée lesion of the thigh (A) Preoperative image showing a fluctuant swelling of the lateral thigh, suggestive of a Morel-Lavallée lesion; (B) Intraoperative exposure revealing seroma evacuation and dissection of the subcutaneous plane; (C) The iliotibial (IT) band appears white and glistening, consistent with chronic fibrotic change; (D) A thick fibrous capsule is identified and dissected, confirming the chronicity of the lesion.

At four weeks post-injury, open debridement and capsulectomy were performed. With the patient in the lateral decubitus position, a 10 cm incision was made at the most fluctuant site. Upon incision, a large amount of serous fluid was released. Dissection revealed a thick fibrous capsule and the iliotibial (IT) band, which appeared white and glistening in texture, consistent with chronic inflammation and fibrosis (Figure 1). A wide cavity was observed extending from the buttock to the proximal thigh.

An extensive capsulectomy was attempted to remove the fibrous lining. However, in the proximal buttock region, the boundaries of the capsule were indistinct and poorly defined, limiting complete excision. Curettage was performed around the IT band to remove residual fibrotic tissue. The wound cavity was irrigated with saline.

The fascia and subcutaneous fat were approximated and repaired with Vicryl sutures in multiple layers to minimize dead space, although complete obliteration of the cavity was not achievable due to its extent and anatomical configuration. A 400 cc Hemovac drain was inserted for postoperative drainage.

Drain output remained at 90-100 cc/day during the first postoperative week. Despite reduced suction pressure, the drain continued to produce 70-80 cc/day. On postoperative day 14, with a persistent output of 80 cc, the drain was removed, and a compression dressing was applied to mitigate infection risk.

Fluctuation recurred, and the patient continued to report discomfort, prompting consideration of an additional intervention. As the initial surgical management was deemed unsuccessful, sclerotherapy was selected as the next step. Doxycycline, a commonly used sclerosing agent, was unavailable in liquid form; although oral tablets were available, converting them into a suitable injectable formulation posed a significant risk of contamination and was therefore not used. Talc, another frequently used agent, was also not readily available. Consequently, sclerotherapy was planned using 10% povidone-iodine (10 ml), 1% lidocaine (5 ml), and saline (5 ml).

**Figure 2:**
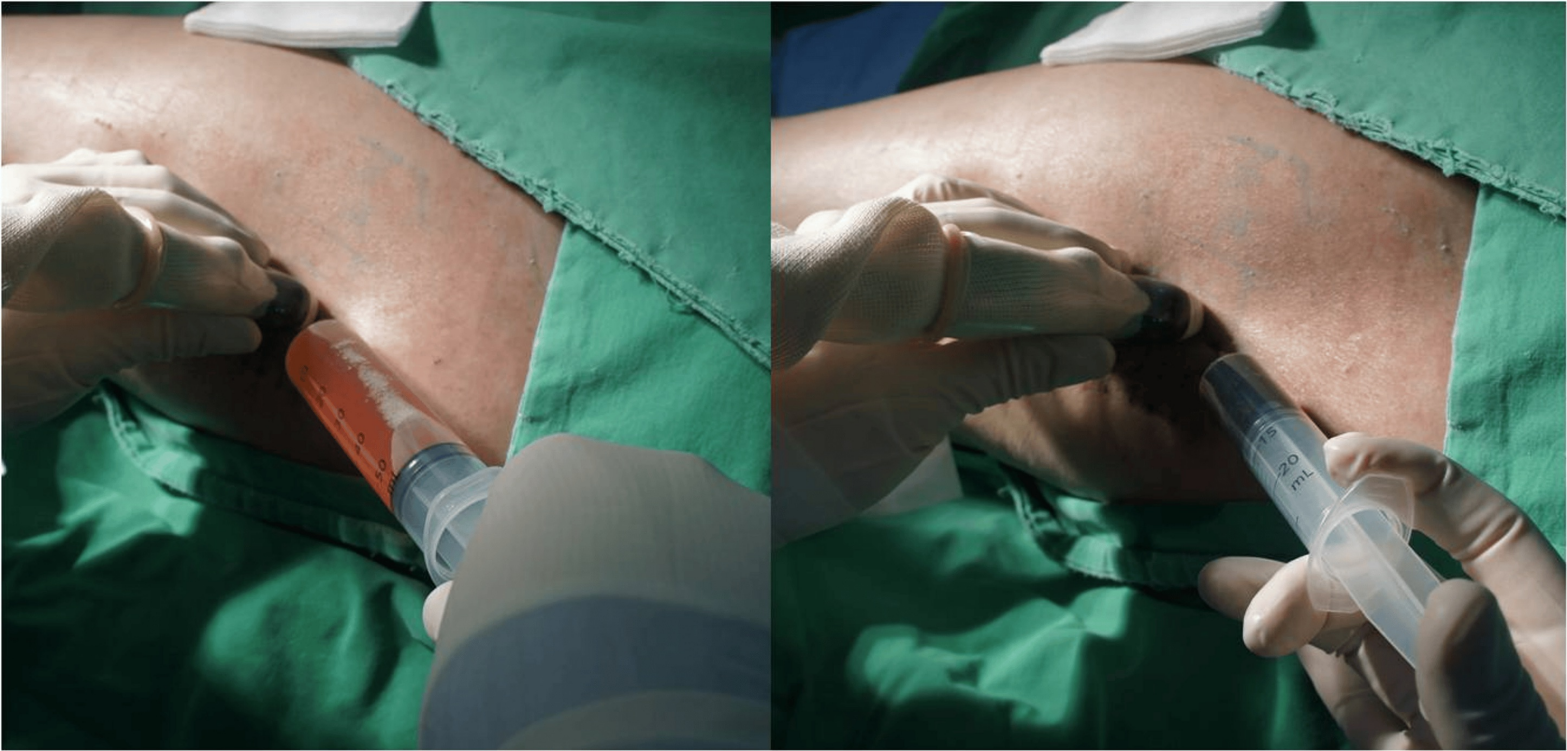
Ultrasound-guided sclerotherapy for recurrent Morel-Lavallée lesion Ultrasound-guided aspiration was performed at four weeks postoperatively, yielding approximately 200 mL of serous fluid (left). Subsequently, the prepared sclerosant solution (povidone-iodine, lidocaine, and saline mixture) was injected through the same needle and retained within the cavity without reaspiration (right), aiming to induce a fibrosing inflammatory response and obliterate the dead space.

Ultrasound-guided aspiration was performed at four weeks post-op, removing approximately 200 cc of fluid (Figure 2). The prepared sclerosant was injected through the same needle and left in place without reaspiration, as the volume of the injected agent was relatively small compared to the size of the cavity, making reaspiration impractical. No signs of infection were noted, and the patient reported only mild discomfort.

After a temporary improvement, the seroma recurred. A second sclerotherapy session was performed two weeks later. During ultrasound-guided aspiration, free fatty material repeatedly obstructed the needle tip, preventing complete aspiration of the seroma fluid. Despite this, the sclerosant was injected into the partially aspirated cavity. This time, reaspiration was performed 40 minutes later to remove the sclerosant-treated seroma contents. Compression dressing was maintained.

At one-month follow-up, the patient reported marked improvement, and ultrasound showed a substantial reduction in fluid collection. By approximately four months postoperatively, although the patient reported some residual discomfort at the surgical site, no recurrent fluid collection was noted on physical examination.

## Discussion

The MLL is a closed internal soft tissue injury caused by traumatic shearing forces that create a potential space between the subcutaneous fat and the underlying fascia. This space subsequently accumulates hemolymphatic fluid and liquefied fat. While acute MLLs may resolve spontaneously, chronic lesions frequently develop a fibrous pseudocapsule, which significantly reduces their responsiveness to conservative management [1,3,11].

Our case demonstrates the clinical course of a post-traumatic MLL located in the gluteal and proximal thigh region, which initially presented without fracture but progressed to persistent seroma formation despite aspiration and corticosteroid injection. The presence of a fibrotic capsule, as identified intraoperatively, is consistent with findings in chronic MLLs and contributes to high recurrence rates even after surgical drainage [3,4,12].

While open capsulectomy and meticulous dead space closure remain mainstays in the management of chronic MLLs [13], complete excision may not always be feasible due to anatomical constraints. Our patient continued to exhibit significant drainage postoperatively, prompting consideration of adjuvant therapies. In such cases, sclerotherapy is a reasonable and minimally invasive option. Doxycycline, a widely studied sclerosant, has demonstrated high efficacy in achieving cavity obliteration [5-7]. However, the unavailability of injectable doxycycline and contamination risks associated with tablet preparation limited its use in our case.

Povidone-iodine, though less commonly used than doxycycline or talc, has demonstrated effective sclerosing properties by inducing a fibrosing inflammatory response in seroma cavities [14]. Sanjeeviah et al. [8] reported a 93% resolution rate in patients with persistent postoperative seromas using 10% povidone-iodine, administered at 20 mL twice daily for up to eight days, with no complications and only one recurrence that resolved with aspiration. Ismail et al. [9] described a case of a recurrent MLL in the lateral thigh that initially underwent open debridement but recurred multiple times and required repeated percutaneous aspirations. Povidone-iodine was eventually used as a sclerosing agent via percutaneous irrigation, resulting in complete resolution without recurrence or adverse effects during a five-month follow-up.

In our case, we adopted a two-stage sclerotherapy protocol using a mixture of 10 mL of 10% povidone-iodine, 5 mL of 1% lidocaine, and 5 mL of normal saline. Lidocaine was added to reduce procedural discomfort, and normal saline served to dilute the iodine concentration, minimizing cytotoxicity. This combination follows previously reported protocols used in pleurodesis to improve patient tolerability. 

This case suggests that povidone-iodine may be a practical and potentially effective alternative sclerosant, particularly in situations where conventional agents are unavailable. However, further studies with larger case series and longer follow-up are needed to confirm its efficacy.

## Conclusions

This case demonstrates the successful use of povidone-iodine sclerotherapy in managing a recurrent posttraumatic MLL after failed conservative and surgical interventions. When standard sclerosants such as doxycycline or talc are not available, povidone-iodine may offer a safe, effective, and readily accessible alternative. Its proinflammatory and fibrosing properties make it a viable option for cavity obliteration in chronic seromas. Further studies are warranted to validate its efficacy and establish standardized protocols for its use in MLL management.
